# Technical, Anatomical, and Functional Study after Removal of a Symptomatic Cavernous Angioma Located in Deep Wernicke's Territories with Cortico-Subcortical Awake Mapping

**DOI:** 10.1155/2013/835029

**Published:** 2013-06-24

**Authors:** Silvio Sarubbo, Gianpaolo Basso, Franco Chioffi, Edward Cesnik, Beatrice Paradiso, Enrico Grandi, Enrico Fainardi, Valeria Tugnoli, Marco Farneti, Enrico Granieri

**Affiliations:** ^1^Department of Neurosciences, Division of Neurosurgery, “S. Chiara” Hospital, 9 Largo Medaglie d'Oro, 38122 Trento, Italy; ^2^Department of Medical and Surgical Sciences of Communication and Behavior, Clinics of Neurology, “S. Anna” University Hospital, 8 Via Aldo Moro, 44124 Ferrara, Italy; ^3^Department of Cognitive Sciences and Education (DiSCoF), University of Trento, 101 Via delle Regole, 38123 Trento, Italy; ^4^Department of Diagnostic and Experimental Medicine, Section of Human Anatomical, Histological and Cytological Pathology, “S. Anna” University Hospital, 8 Via Aldo Moro, 44124 Ferrara, Italy; ^5^Department of Neurosciences and Rehabilitation, Division of Neuroradiology, “S. Anna” University Hospital, 8 Via Aldo Moro, 44124 Ferrara, Italy; ^6^Department of Neurosciences and Rehabilitation, Neurophysiology Unit, “S. Anna” University Hospital, 8 Via Aldo Moro, 44124 Ferrara, Italy; ^7^Department of Neurosciences and Rehabilitation, Division of Neurosurgery, “S. Anna” University Hospital, 8 Via Aldo Moro, 44124 Ferrara, Italy

## Abstract

*Introduction.* The subcortical region underneath Wernicke's area (WA) is a critical crossing of the eloquent language pathways involved in all semantic, phonological, syntactic, and working memory elaboration. We report the resection of a CA located underneath the dominant WA discussing the functional and anatomical evidence provided by fMRI, dissections with Klingler's technique, and intraoperative mapping during awake surgery. *Case Report.* A 64-year-old right-handed female affected by daily complex focal seizures underwent f-MRI, showing language activations in the middle and inferior temporal gyri and an unusual free entry zone in the “classical” WA. The cortical intraoperative mapping partially confirmed the f-MRI results, and we approached the lesion directly through WA. Subcortical DES allowed the identification of the eloquent language pathways and the radical resection of the perilesional gliotic rim. The patient did not report deficits and she is seizures and drug free after 1-year surgery. *Discussion*. Cortical DES demonstrated the variability of the eloquent areas within the cortex of the dominant temporal lobe. The subcortical DES confirmed the crucial role in language elaboration and the anatomical course of the bundles underneath WA. *Conclusions.* Awake surgery with DES represents a reliable and dynamic technique also for safer and functional-customized resection of CAs.

## 1. Introduction

**Figure 1:**
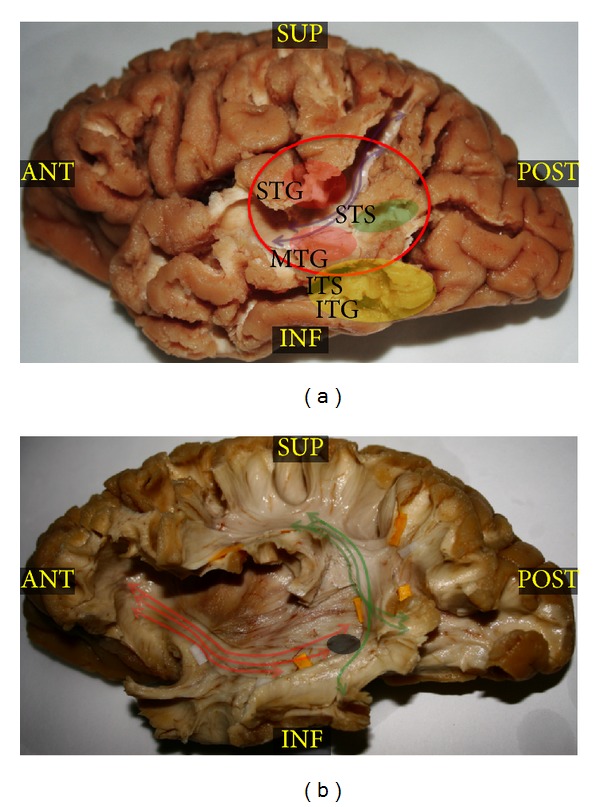
(a) This left hemisphere dissected with Klingler's technique is focused on the posterior temporal lobe after removal of the gray matter of the middle and posterior thirds of the superior and inferior temporal sulci (resp., STS and ITS) and of the inferior parietal lobule. The purple arrows show the direction of the indirect posterior portion of the superior longitudinal fascicle (SLF), connecting the inferior parietal lobule (angular and supramarginal gyri) to the Wernicke's territories (posterior thirds of superior and middle temporal gyri and superior temporal sulcus, BA 22), which are evidenced by the red circle. The red circles at the level of the junction between the posterior and middle thirds of the superior and middle temporal gyri (resp., STG and MTG) represent the eloquent sites identified by means of cortical DES during denomination task. The yellow circle identifies the inferior temporal gyrus (ITG) language areas shown by fMRI and confirmed by means of cortical DES. Finally, the green circle shows the cortical approach to the lesion directly through the posterior third of the STS (BA 22). (b) After removal of the more superficial cortices of the STG and MTG, the courses and terminations of the direct component of the SFL, the arcuate fasciculus (AF), are demonstrated and evidenced by green arrows. Red arrows evidence the more ventral fibers of IFOF, crossing the AF just underneath the Wernicke's territories. The grey circle shows the position of cavernous angiomas (CA) in respect to the deeper bundles. ANT: anterior; INF: inferior; ITG: inferior temporal gyrus; ITS: inferior temporal sulcus; MTG: middle temporal gyrus; POST: posterior; STG: superior temporal gyrus; STS: superior temporal sulcus; SUP: superior.

The prevalence of central nervous system (CNS) cavernous angiomas (CAs) ranges between 0.02 and 0.5% in the population, and they account for the 5–10% of all cerebro-vascular malformations (CVMs) [1–3]. These lesions usually present between the 3rd and 5th decades of life [4], the patients experience seizures in 50–70% of cases [5–8], and the natural history of CAs is characterized by growth and/or micro or macrobleeding [5, 9–12]. According to the literature, microsurgical removal of supratentorial CAs allows a seizures' reduction in 70–100% of cases [13, 14]. In case of a single symptomatic CAs, with microbleeding signs and located in high eloquent area, the microsurgical removal seems reliable and effective in order to avoid the potential risks due to a future macrobleeding, growth or chronic seizures [5, 15]. However, CAs located in eloquent cortico or subcortical regions represent a real challenge for the neurosurgeon for at least three reasons. Firstly, CAs' resection is frequently not sufficient to obtain the best postoperative seizures control. Even if still debated, in fact, the removal of perilesional hemosiderin gliotic rim seems crucial to reach the best control of seizures [3, 15–20], and the common mini-invasive approach is not always sufficient for the best outcome. Secondly, if subcortical CA is located in regions with high eloquent fiber pathways crossing, eventual lesion of white matter bundles will produce permanent deficits, because plasticity has not been demonstrated for human white matter. Finally, the safest cortical approach to the deep-sited CAs is not always predictable, especially in case of locations underneath high variable eloquent regions, such as Wernicke's area (WA, i.e., the posterior thirds of the superior and middle temporal gyri and superior temporal sulcus, BA 22) (Figure 1(a)).

 WA was classically considered the area of the language “comprehension,” deputed to the semantic content of the sensitive and motor elaborations of the language [21]. However, the advent of the most advanced neuroimaging techniques (i.e., fMRI, MEG, etc.) and the intraoperative direct electrical stimulation (DES) studies demonstrated a more complex role and high variable interindividuals distribution of eloquent sites in this area [22–25], according to the modern hodotopical organization of brain functions (i.e., distributed neural subpopulations with different roles in functional elaboration, sited also at long distance and strongly connected by means of high-specialized bundles subserving parallel large and parallel working networks). Different subregions in WA were, in fact, demonstrated to be involved in different networks of language elaboration. Phonological, syntactic, and semantic activations were, in fact, evidenced in this area [22], and it was recently attributed to the more appropriate definition of “Wernicke's territories” [26]. Moreover, the subcortical region underneath WA represents a crucial crossing site (Figures 1(a) and 1(b)) between the most important eloquent language bundles and particularly the posterior indirect portion of the superior longitudinal fascicle (SLF), connecting the WA to the Geschwind territories (i.e., angular gyrus and supramarginal gyrus of the inferior parietal lobule) (Figure 1(a)); the arcuate fascicle (AF), direct component of the SLF, connecting WA to the inferior frontal gyrus (IFG) (Figure 1(b)); and the inferior fronto-occipital fasciculus (IFOF), connecting the superior parietal lobule and occipital striate and extrastriate cortices to the frontal lobe (Figure 1(b)). These bundles subserve, respectively, the language perception (namely, the syllable discrimination and identification), the dorsal phonologic and the ventral semantic networks, and terminate within and cross underneath Wernicke's territories (Figures 1(a) and 1(b)). For these reasons WA was considered for a long time a nonremovable region. However, the recent technical advances in awake craniotomy (i.e., intraoperative brain mapping and neuropsychological monitoring) and in neurosciences allowed surgical approach also in this high-eloquent region, demonstrating also new insights into language networks organization and neural plasticity [27–32].

**Figure 2:**
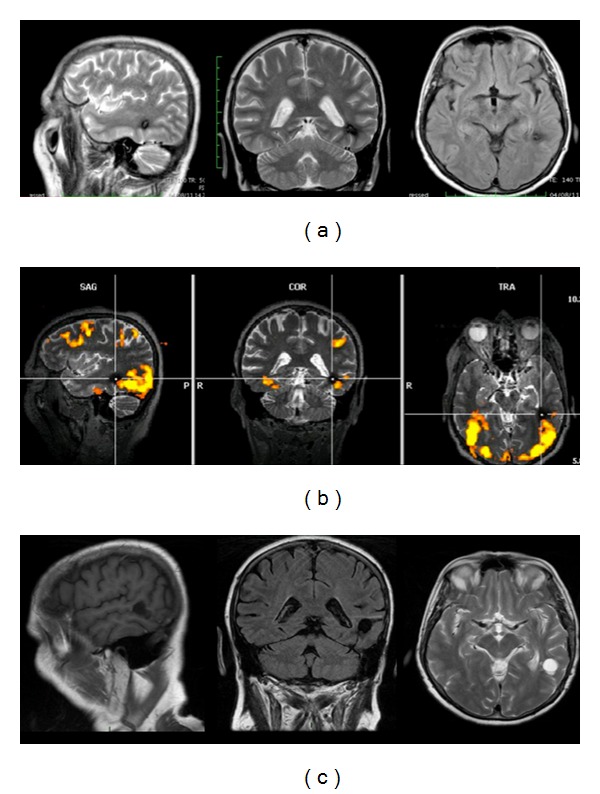
(a) Preoperative MRI sequences (sagittal T2, coronal T2, and axial Flair) showing a 1 cm mixed-intensity lesion with a hypointense rim into the subcortical region of the middle temporalgyrus (MTG) directly in contact to the stems and/or terminations of the subcortical language pathways of this region (i.e., inferior fronto-occipital fasciculus, arcuate fasciculus, and indirect posterior component of superior longitudinal fascicle). (b) The preoperative 4T f-MRI (MR Scanner MedSpec, Bruker) showing the main activation during language task of the posterior temporal lobe on sagittal, coronal, and axial planes. The f-MRI demonstrated significant activations at the level of the posterior thirds of inferior temporal gyrus (ITG) and MTG in the BA 37 and in the occipital laterobasal cortex in the BA 19. No activations were detected at the level of the classical Wernicke's area at the level of the posterior thirds of the MTG and superior temporal gyrus (STG) and perisuperior temporal sulcus (STS) in the BA 22. (c) The postoperative MR sequences (sagittal T1, coronal Flair, and axial T2) demonstrated the complete resection of the cavernous angiomas (CA) and of the perilesional hemosiderin gliotic rim. CA: cavernous angiomas; ITG: inferior temporal gyrus; MTG: middle temporal gyrus; STG: superior temporal gyrus; STS: superior temporal sulcus.

Here, we report a case of removal of a symptomatic CA located in deep left dominant Wernicke's region (Figures 1(b) and 2(a)) during awake surgery with cortico-subcortical DES and electrocorticographic monitoring. We compared the functional evidence provided by preoperative 4T f-MRI, intraoperative mapping, and white matter dissection of this region with Klingler's technique, discussing how all of these influenced the surgical approach and the clinical result and proposing a customized functional approach for deep-sited CAs.

## 2. Case Report and Operative Technique

### 2.1. History

A 64-year-old right-handed woman (according to the Edinburgh Handedness Inventory Score and preoperative f-MRI) visited our hospital complaining of daily complex focal seizures with language disturbances (transient semantic paraphasias and anomias) and vertigo. The T2-weighted MRI showed a mixed-intensity 1 cm lesion with a hypointense rim underneath the border between the middle and posterior thirds of middle temporal gyrus (MTG) (Figures 1(a) and 2(a)), directly in contact with the subcortical language pathways (particularly IFOF, AF, and indirect posterior portion of SLF) (Figure 1(b)).

### 2.2. Examination

Standard neurological examinations, MMSE, Laiacona-Capitani denomination test, Token test, and phonological and semantic verbal fluency were collected 1 day before surgery and 1 week, 3 and 6 months, and 1 year after surgery. Preoperative Laiacona-Capitani score was 77/80; MMSE, Token test, and semantic and phonological fluency were normal. A preoperative f-MRI (4 Tesla MR scanner, MedSpec, Bruker) revealed diffuse cortical activations during denomination task (Laiacona-Capitani denomination test) in the posterior third of left MTG and middle and posterior thirds of the inferior temporal gyrus (ITG) (Figures 1(a) and 2(b)). Considering bleeding risk in eloquent region and the epileptic seizures, we proposed to the patients the surgical resection in awake surgery.

### 2.3. Surgery

The patient underwent completely awake surgery (no intubation was performed and Remifentanil and Propofol infusion was stopped at dura opening) with intraoperative cortico-subcortical DES (7 mm-spaced bipolar stimulator), during Laiacona-Capitani denomination test and counting test (from 0 to 10) and electrocorticography (in order to avoid false positive sites at stimulation). We used intraoperative neuronavigation (Stealth Station, Medtronic, Minneapolis, MN, USA) to define the exact location of the CA and the best trajectory to reach that with minimal manipulation of healthy cerebral tissue and according to the results of DES.

**Figure 3:**
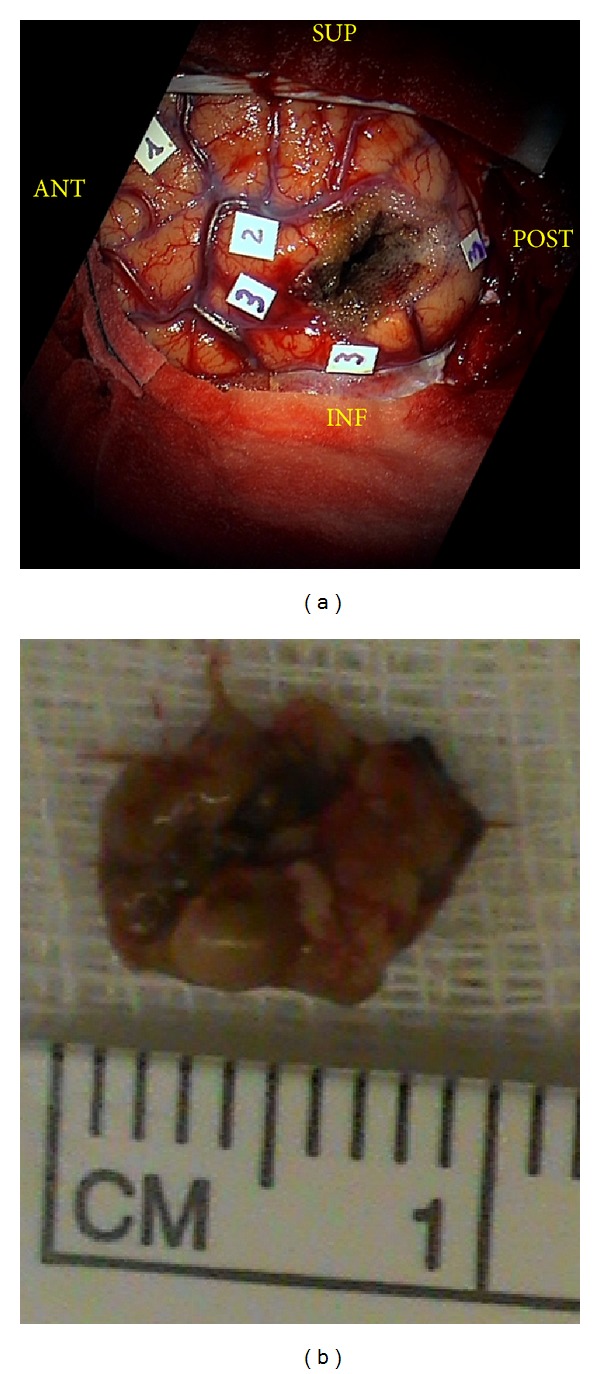
(a) The intraoperative picture after resection shows the results of the direct electrical cortical mapping. The stimulation threshold was set at 3 mA after eliciting speech arrest at stimulation of ventral premotor cortex (VPMC) during counting task (tag 1). The cortical stimulation during denomination test (Laiacona-Capitani) elicited semantic paraphasias (tag 3) at the level of the posterior thirds of the inferior temporal sulcus (ITS) and middle temporal gyrus (MTG; BA37 and BA21), at the extreme posterior third of the superior temporal sulcus (STS), posteriorly to the lesion, and at the border between middle and posterior thirds of the STS and superior temporal gyrus (STG; BA22) anteriorly to the lesion. Anomias (tag 2) were elicited at the border between middle and posterior thirds of the STG (BA22) at the upper and anterior margin of the lesion. The site of corticectomy was in the free activations zone in the posterior third of the peri-STS area (BA22). In the deepest part of the surgical cavity direct electrical stimulation elicited semantic paraphasias due to deactivation of the inferior fronto-occipital fasciculus (IFOF). (b) This picture shows the 1 cm cavernous angioma (CA) removed. BA: Brodmann area; IFOF: inferior fronto-occipital fasciculus; ITS: inferior temporal sulcus; VPMC: ventral premotor cortex; STG: superior temporal gyrus; STS: superior temporal sulcus.

The stimulation threshold was set at 3 mA after eliciting speech arrest at stimulation of ventral premotor cortex (VPMC) during counting task (Figure 3(a), tag 1). The cortical DES during denomination task elicited systematically semantic paraphasias at the level of the cortical projection of the lower margin of the lesion (i.e., posterior thirds of the inferior temporal sulcus and MTG) (Figures 1(a) and 3(a)), posteriorly to the lesion (extreme posterior third of the superior temporal sulcus) and anteriorly to the lesion (i.e., at the border between middle and posterior thirds of the superior temporal sulcus and superior temporal gyrus) (Figures 1(a) and 3(a)); anomias at the level of the cortical projection of the upper and anterior margin of the lesion (i.e., at the border between middle and posterior thirds of the superior temporal gyrus) (Figures 1(a) and 3(a)). Considering the results of cortical stimulation we choose the posterior third of STS (BA22) to approach the lesion (transsulcal approach). We reached the CA in the deep white matter at 3 cm from the cortical surface. After the complete resection of the CA (Figures 1(b) and 3(b)), we performed bipolar stimulation during denomination task and we completed the resection of the perilesional hemosiderin gliotic rim by suction and up to the subcortical functional limits, constituted in this case by the evocation of semantic paraphasias at DES of the IFOF in the most inferior and medial portion of the surgical cavity. The total duration of surgery was 3 hours.

### 2.4. Postoperative Treatment and Course

The patient did not report any postoperative deficit, even transient. She was discharged the second morning after surgery and came back to her normal socioprofessional life two weeks after surgery. Over 1-year followup (1 week, 3 and 6 months, and 1 year after surgery), Laiacona-Capitani score improved (80/80) and the other tests results were unchanged. The patient is actually seizures and drug free. The postoperative MRI (3 months after surgery) demonstrated the complete resection of CA (Figure 2(c)).

### 2.5. Anatomical Dissection

A fresh left hemisphere was prepared according to the Klingler's technique (i.e., fixed in formalin solution 10% and successively frozen at −20°, as previously described by the first author) within the Project for Human White Matter Study of the Ferrara's University and as approved by the “S. Anna” University Hospital Ethical Committee. The microscopic dissection was performed with wooden spatulas starting from the cortices of the temporal and parietal lobes and proceeding to the frontal ones. After complete removal of the gray matter of the main temporal, parietal, and frontal sulci, preserving the gray matter on the top of the gyri for the terminations' study, the main U-fibers were exposed and successively removed in order to demonstrate the main associative pathways of the perisylvian region, proceeding in a lateromedial direction: indirect posterior portion of SLF, anterior indirect portion of SLF, AF and, finally IFOF. Sequential pictures were captured all over the different steps of the dissection, according to our protocol.

## 3. Discussion

The goals of CAs' surgery in eloquent region are the prevention of bleeding (or rebleeding) and growth and the resolution of eventual symptoms. The annual bleeding risk for these lesions ranges from 1.6% to 2.6% [3, 33, 34]. Thus, CAs located within eloquent white matter are possible cause of high neurological morbidity. Moreover, the complete resection of the pericavernomatous hemosiderin gliotic rim is considered crucial for the best epileptic outcome. The perilesional area is known, in fact, to contain potential-associated telangiectasias or microscopic satellite cavernomatous lesions carrying a risk of rebleeding [35, 36], and the hemin deposits and hemosiderin fringe due to CA's microhemorrhage are considered to be responsible for CAs' associate epilepsy [20, 37]. For this reason, in case of seizures, the sole and mini-invasive resection of the CA could not be effective for the complete resolution of symptoms [38], and different authors demonstrated the crucial impact of the removal of perilesional gliosis on the final outcome [15–19, 39]. However, sometimes a radical resection can be extremely dangerous and it is avoided, especially in case of CAs deeply sited in eloquent subcortical white matter.

Awake surgery was largely demonstrated to be a reliable technique for removal of cerebral tumors [27–30] in eloquent areas and also a valid tool for the “in vivo” study of the brain functions and networks [23, 28, 30, 31]. Nevertheless, solely 15 cases (in 5 papers) [3, 40, 41] have been reported in literature about resection of CAs in critical language areas with cortico-subcortical DES and no specific descriptions have been provided about functional organization and anatomical connectivity of Wernicke's cortico-subcortical regions in these cases. In the case we report, considering the high eloquent site of CA, the risk of dramatic and permanent deficit due to eventual hemorrhages and the daily epileptic seizures, we proposed to the patient the lesion resection with awake surgery monitoring.

We performed a preoperative 4T f-MRI in order to obtain a wide picture of the language network's organization for the best surgical planning. The 4T f-MRI showed crucial activations during denomination task at the level of middle and posterior thirds of IFG and posterior third of MTG (Figures 1(a) and 2(b)). During awake surgery the cortical intraoperative DES confirmed the data of the f-MRI (Figures 1(a) and 3(a)) but demonstrated also crucial and expected eloquent sites at the level of the junction between the middle and posterior thirds of MTG and STG (Figures 1(a) and 3(a)). Abruptly, we did not find eloquent sites during language task at the level of the more “classical” Wernicke's territories (i.e., middle and posterior thirds of the STG and MTG and peri-STS) (Figures 1(a) and 3(a)). Thus, we decided to perform the corticectomy in WA, directly through the STS (Figure 1(a)), approaching the lesion from a more ventral and superior entry point than normally expected and planned (Figures 1(b) and 2(c)). The results of preoperative f-MRI confirmed the high sensitivity of this imaging technique but also the limitations (i.e., lower specificity and spatial resolution) about its use for neurosurgical purposes [42] as alternative to DES. Even if cortical DES confirmed the more ventral temporal activations (Figures 1(a) and 3(a)), in fact, it showed also unexpected more dorsal eloquent sites (Figures 1(a) and 3(a)) and led us to an approach classically considered “unsafe” for functional preservation (i.e., trans-STS; BA 22), just posteriorly to these sites (Figures 1(a), 2(c) and 3(a)). The results of awake mapping provided evidence more of the extreme interindividuals variability of the distribution of language eloquent sites, even in cortices crucial for functional elaboration such as WA. Moreover, DES evoked both semantic paraphasias (at the level of MTG) (Figures 1(a) and 3(a)) and anomias (at the level of the STG) (Figures 1(a) and 3(a)), further supporting the complex and multimodal role of Wernicke's territories in language elaboration, as suggested also by other authors. Finally, it is worth noting that a direct approach trans-WA (BA22) was performed to remove an intracerebral deep lesion without functional impairment, demonstrating once again that the individual functional assessment of each patient is more crucial than the classical functional-anatomic landmarks, in order to tailor a safe and radical surgery. Moreover, after isolation and removal of the lesion we performed subcortical stimulation DES (not evoking language disturbances), in order to avoid eventual damage to IFOF, AF, or SLF bundles or their terminations. Considering the results of this first subcortical stimulation and also the epileptic symptoms of the patient, we decided to remove also the hemosiderin gliotic rim up to visualize normal brain tissue and to identify the functional subcortical limits. Beyond the perilesional gliotic rim, the subcortical DES evoked semantic paraphasias in the most ventral and inferior portion of the surgical cavity. The anatomical site of stimulation and the functional impairment it evoked (i.e., semantic paraphasias) are, definitely, according to the anatomical course of the IFOF in this region (Figure 1(b)) and support the functional role that was recently attributed to this bundle as main ventral semantic stream [31, 32].

Finally, the postoperative 3-month MRI demonstrated the complete resection of the CA and the perilesional hemosiderin gliotic rim (Figure 2(c)). The total duration of the surgery was 3 hours. The patient well tolerated the surgical procedure and she did not experience postoperative worsening or further seizures. She was discharged just after 48 hours and over the followup her language performance improved. After 1-year followup she is drug free and she never experienced further seizures.

## 4. Conclusions

DES is the most sensitive and specific tool for identification of each individual cortical functional assessment. The distribution of the functional cortical sites involved in language elaboration is extremely variable, even in high eloquent regions such as WA, and no regions should be considered “a priori” nonapproachable or nonremovable.

This report supports the role of intraoperative cortico-subcortical DES such as an extremely reliable and specific technique also for safer, radical, and functionally tailored resections of CAs located in cortical and/or subcortical eloquent regions.
